# Three Families Poisoned by Eating Ice Cream, at Macon, Ga.

**Published:** 1878-02

**Authors:** William A. Greene

**Affiliations:** Macon, Ga.


### THREE FAMILIES POISONED BY EATING ICE CREAM AT MACON, GA.—THE SOURCE OF THE POISON, AND REPLY TO THE OPINIONS OF DR. C. A. CHEATHAM, PUBLISHED IN THE "DAWSON JOURNAL," AND DR. MATTAUER, AS HERETOFORE REPORTED IN THE "MACON TELEGRAPH AND MESSENGER."

---

BY WILLIAM A. GREENE, M.D,, MACON, GA.

---

In the month of September, 1877, there occurred in this city a serious accident of poisoning from eating ice cream, in a boarding-house kept by Miss Gregory, in which some fifteen or more persons were the victims. The affair created a great excitement in the community, and various and conflicting reports were spread abroad as to its cause and origin. Four members of the family of Dr. C. A. Cheatham, of Dawson, Ga., were among the number thus poisoned, and his daughter, a beautiful and accomplished young lady, died within a month, and before this excitement had subsided another family were attacked in precisely the same manner, as well as a neighboring family to whom a portion of the ice cream had been sent. Of course the anxiety and excitement of the community were greatly increased by this second accident. Mr. James Seymour, in whose family this last occurrence took place, and who had been an invalid for several years, and also partook of this ice cream, also died. Our distinguished fellow townsman, Dr. Mattauer, was the attending physician of this family. Very soon after the first occurrence, in Miss Gregory's boarding-house, Dr. Cheatham published an account of the cases in his own family, and gave his views as to the cause, origin, and treatment, which was published in the Dawson *Journal*, a newspaper of his city and home. And after the second occurrence, the reporter of the local column of the Macon *Telegraph and Messenger* called on Dr. Mattauer, and published in his paper an account of the interview, quite fully. These gentlemen did not differ materially in their opinions. I do not deem a newspaper the proper channel for the discussion of subjects in medicine, and, therefore, ask space in your JOURNAL for the investigation of the causes of these unfortunate accidents. A large number of physicians in this portion of Georgia, no doubt read these newspaper articles, and will be better prepared to understand the points I make in attempting to controvert their positions, and, without consuming too much space, I hope to make myself sufficiently intelligible to all. It will be seen the non-professionals have taken much interest in this matter; but my article is not intended to make any impression in *that* direction, but to arrive at, if possible, the true solution of the mystery. I would be pleased to hear from either of these eminent gentlemen again, for whose opinions on subjects in the profession I have the profoundest respect. I will not attempt to correct the inaccuracies and errors in the statements of

Dr. Cheatham, in the Dawson *Journal*, as to the history of the treatment and management of the cases of his own family, who were among the unfortunate victims of poison, as many who were present and witnessed the conduct of the cases well know how imperfect is the Doctor's recital of what occurred. Suffice it to say, he wrote what he *believed* to be true, and any controversy in this direction would be inappropriate, besides, intruding on the sacredness and sanctity of grief and mourning, and bring back thoughts and reflections which had better be buried with the "dead past," as the living could not be benefited, and it is better that the ashes of our departed ones rest in peace.

That two deaths resulted from the poison, admits of no question, whether direct or indirect, and that the ice cream was the vehicle conveying this poison is equally as palpable. None deny this.

Then, since this is a delicacy and luxury which nearly all the human race partake of during certain months of the year, and almost daily, must increase the necessity and anxiety for a most critical examination of the probable or possible reasons why this particular ice cream should have been the source of such fatal consequences. In Europe, or perhaps in any of our Northern cities, such an occurrence would never have been permitted to pass by with so little apparent interest to discover the true cause of the accident.

Anything, or any substance, taken into the stomach capable of producing the prompt and violent symptoms that occurred in those fifteen or twenty persons affected, must be out of the order of simple causes, which brings us to the discussion and investigation of the subject under review.

At a certain boarding-house in the city of Macon, within three or four hours after partaking of the usual dinner, all who were present, except one or two who failed to eat ice cream which was served, were suddenly prostrated with violent symptoms, as cramps, nausea, and severe vomiting and purging, extreme pain in stomach and bowels, with dryness and thirst, suffused and watery eyes, and in two cases delirium. Great excitement ensued, and friends and physicians were rapidly summoned to their bedsides. Within a month, in the same vicinity, from precisely the same cause, (eating of ice cream) another family are attacked almost in the same manner, as well as a neighboring family to whom a portion of the ice cream had been sent.

We must necessarily meet with many perplexities in the solution of the questions bearing on cases of sudden poisoning under such circumstances, whether viewed from the standpoint of medico-chemical investigation or of its medical jurisprudence. But by the aid of correct chemical knowledge we will certainly find a correct solution for all doubts; for our acquaintance with our weapons of defense against all disease, or abnormal conditions, depend for their very existence upon a knowledge of chemistry. In chemistry mere hypotheses have no existence, everything must be subjected to experiment; the re-agent and the crucible, the blow pipe and the test, are weapons of argument, and from them there is no appeal. The beautiful simplicity of its laws, admitting of no doubt or confusion, the certainty of its results, must enchain the reason of all who become introduced to them.

Being called to the cases in the house of Miss Gregory, I carefully watched and noted every symptom and the progress of the cases. I was troubled at the time for a solution of the cause of so urgent and alarming symptoms as above mentioned. They evidently embraced the ordinary symptoms of some corrosive or irritant poison, and it is the rule in such emergency, when the physician cannot take the time to inquire into the history of the attack, and investigate everything required to arrive at a correct opinion, to treat the symptoms before him as indicated, the most prominent being the agonizing pain, the incessant vomiting and coliquative perspiring—which seemed to be threatening life—and to apply such antidotes as seemed probably necessary. This was hurriedly done, and the patients made comfortable. Then begun the examination, so far as facilities were furnished for investigating the causes. Feeling my own inefficiency as an expert toxicologist for such work, I at once began a correspondence with the best chemists and toxicologists in the country, and examination of the best authors, being satisfied the opinions advanced by Dr. Cheatham in the article referred to, as well as the reported ones of Dr. Mattauer, were utterly untenable. Therefore the cause must be sought for somewhere else, as there was no suspicion of criminal intent. There have been in the community various and conflicting reports of the professional opinions of the medical gentlemen in attendance, and the non-professionals have very learnedly (!) discussed these opinions and given *their* dictum as to their correctness, value and validity. The truth is, the duties and responsibilities of a physician, when called to cases of sudden and suspected poisoning, are very delicate and embarrassing, and it is necessary he should know to what points he ought to give his attention. While giving medical advice and as far as possible relieving anxiety, it is in his power to perform other duties, if the case should be one of suspected criminal poisoning. In the excitement of the occasion many facts of importance are overlooked. The physician is not expected to make himself too officious on such occasions, but would be unmindful of his duty as a member of society, if he did not aid the cause of justice by extending his scientific knowledge to the detection of crime. But we cannot, in a paper like this, discuss the duties of a medical jurist in cases of suspected poisoning, but refer to the various works on medical jurisprudence—prominent among them, and recognized as authority both in Europe and America, is "Taylor on Poisons," edition of 1877—which should be in the library of every physician and lawyer of the land.

*The Danger of Premature Opinions.*—When suddenly called to a case of suspected poisoning, and during the examination, the physician is often pressed to give an opinion respecting its nature, before the steps for a process of investigation are completed. This may arise from the anxiety or curiosity of those who are interested in the case, or by the community at large. There is a rule which ought always to be followed on these occasions, that no opinion whatever should be expressed until an analysis is completed. But it often happens, even in the hands of the best chemists, that the last steps of a process lead to a very different result from what was anticipated at the commencement—a suspicion derived from a few incipient experiments is very likely to be overthrown by continuing the investigation. This occurred with me in these very cases under consideration, when I announced by a hurried and simple test that I found mercury in the ice cream, but afterwards found lead, as did others in examining the same sample, and I offer the following cases, which may be found related in "Taylor on Poisons," as some extenuation for my error and hurried expression of opinion: A celebrated case known as the "Broughton Case," in London, where Dr. Bothray gave an opinion in the first instance that the poison administered to the deceased was arsenic, and subsequently testified death was caused by laurel water. Another case where arsenic was supposed to be administered, sulphuric acid was the poison. Again, in a case tried at the King's assizes, a medical expert admitted that at the coroner's inquest he stated the poison to be arsenic, but by subsequent experiment he found it to be oxalic acid. And many other errors may be found in the old English works on medical jurisprudence. Hence the imprudence of expressing an opinion from a few experiments, or until the analysis is complete.

Modern chemical science convinces us that the experiments of testing poisons by giving the suspected article to animals, as cats, are not fitted to convey to us any accurate knowledge of symptoms or doses required to produce fatal effects, and cannot be depended upon; in fact, are perfectly unscientific and unreliable. The "reporter" must remember that his conclusions must be based only on medical facts, not upon novel circumstances. Nor should information derived from others be the basis of an opinion by a medical man, and it is scarcely necessary to remark that a conclusion based upon mere probabilities is of no value as evidence, hence should never be given as such. These rules have always been observed by the best writers, but are totally disregarded by more recent "chemists" and physicians.

It is unnecessary in this paper to recite well-established rules for discovering the causes and sources of poisoning, in either accidental cases or those from criminal intent, all intelligent physicians being acquainted with them; besides, can be found in works "On Poison and Medical Jurisprudence." But in these cases under consideration, it seems to me all the conditions except the most important ones were noticed in the reports furnished the public by Dr. Cheatham, viz., a chemical examination of the ice cream supposed to contain the poison, and of the matters vomited, and *post mortem* appearances and chemical examination of those who died. No opinion could be worth much without these.

The theory advanced by Dr. Cheatham is not sustained by any authority I can find in any works on the subject, but on the other hand, I find abundant evidence of its fallacy. Suppose it were true that any condition of fermentation possible to take place in the mixture (of ice cream custard) could produce the results in these cases, at any time or under any circumstances, we at once see how dangerous it would be for us to use this delicacy that is so common among the people, as accidents of this nature would be of constant occurrence, in place of being exceptional, as no amount of care could prevent it. We are aware that on all excursions and festival occasions this custard is prepared the over night and kept in some convenient vessel, to be frozen when required for use. This simple illustration at once destroys the possibility of any such condition being probable from fermentation of the custard as to produce symptoms of acute poisoning. It would then certainly be hazardous to make ice cream, one of our most usual dishes on any occasion, as we all are aware it is seldom absent from the "bill of fare." But there is far better authority than this, and of such a character that none acquainted with the source will for a moment question. It is from the celebrated and well-known chemist and toxicologist, Dr. Edward R. Squibbs, of Brooklyn, New York, who has for a long life time made this branch of our profession a special study, and whose opinions on such subjects for twenty years have been acknowledged as the highest authority in America and Europe. He says in a private letter on this subject, written to me: "I cannot believe that any fermentation can occur in any mixture of milk, eggs and sugar, within thirty-six hours, in either tin or earthen vessels, or under any ordinary circumstances, that could at all account for symptoms of acute poisoning in persons who had eaten it—the mixture. There is nothing in the present state of knowledge in the process of fermentation to warrant any such conclusion, nor anything to warrant the supposition that any germs formed in such mixture within the time mentioned, or indeed in any length of time compatible with the mixture being in an edible condition, could be poisonous or be developed into poisonous animalculæ."

After which, on this point, he further asserts:

"You may justly infer from this that the present state of knowledge, so far as I am acquainted with it, will not justify nor in any degree support a conclusion that any mixture of milk, egg, sugar and common salt, flavored with essence, can under any circumstances cause symptoms of acute poisoning while yet in a condition to be tolerated as human food."

Can the use of words or expressions put the case any stronger? This opinion of Dr. Squibbs was written with all the facts before him, as well as the article of Dr. Cheatham and the reported opinion of Dr. Mattauer, as published in the local columns of the Macon *Telegraph and Messenger*, and in perfect accord of my own opinion, as stated at the time to numerous persons of the city, who had very carefully read the statements of both these celebrated physicians as published. Then the question arises, where will we find the true source of the causes of this accident? We must certainly search for it outside of anything found in the fermentation process. One thing we can be sure of—that there is no danger of trouble from "germs" or "microscopic fungi," called by these hideous names, "sarceni ventriculi" and "torula cerviscæ," as the results of "fermentation," either "incipient" or otherwise. Ice cream behaves to-day just as it did in all by-gone ages, when no such creatures were heard of as inhabiting the custard, before, during or after freezing and taking it into the human stomach. But I hope, before concluding, to direct your attention to circumstances and conditions, which, if properly observed, will prevent such trouble as we have witnessed, and perhaps save life, and which will account for the unfortunate accidents in these families. I could produce numerous authorities for sustaining my position, but will cite only one other in this connection.

I also corresponded with S. P. Sharples, M.D., State Assayer of Massachusetts, and chemist at Harvard University, at Cambridge, who says, after carefully studying the published statements of Dr. Cheatham and Dr. Mattauer:

"The explanation of Dr. Mattauer of a well-known fact is hardly tenable, I think, in these cases."

"Taylor on Poison" says milk is subject to be affected by diseases in the cow, and it acts like an emetic, producing vomiting and violent symptoms, and reports three children who ate milk at breakfast, and were attacked with violent vomiting and pain, with blood in the ejected matter, when poison was suspected; but the proper test failed to find any, when it was discovered the milk was from a diseased cow. "Milk cannot possibly contain, by any properties of its own, anything to produce such violent symptoms."

The reporter of the Macon *Telegraph* stated, in the cases of Dr. Mattauer (Seymour's family), that "the fact that the custard in this instance stood overnight in an earthen vessel, not in a tin one, explains the theory of lead poison." Dr. S. says on this point: "The fact of lead poisoning is greatly strengthened. The remark that the custard was kept in an earthen vessel, and therefore there could be 'no lead in it,' I should take decided exceptions to, as lead glaze in earthern vessels is the most common source of poisoning."

I was surprised at the time when this argument was used against the idea of lead poisoning in the first cases. Dr. S. further says, in referring to the cases at Miss Gregory's:

"Another thing I should be very suspicious of, is the lemon flavoring which was used in double quantities,"

which greatly increases the probabilities of lead accounting for all this trouble, and which I shall again notice.

These authorities are sufficient to destroy the theory of "germs" or "microscopic fungi," accounting for the source of the poison as emanating from the process of fermentation. If this were true, these accidents would be of daily, if not hourly, occurrence. It is impossible. Then we say that the lead contained in the custard can alone give a proper solution of the mystery, the second case strengthening this conclusion. Besides, lead was found in the custard of the first case by at least two chemists, but in very small quantities. But we well see what very small and minute portions are referred to produce the most violent symptoms. Unfortunately, as before remarked, the conditions for discovering the causes properly and certainly were overlooked at the time. An inquest would have brought out all the facts. As yet, our knowledge on many of these points is quite imperfect, consequently these cases have excited far more interest than many are aware of among medical men at a distance. As an instance of our imperfect knowledge, we find that poisoning by imperfectly made cheese is well known, though the best authorities are not agreed as to the cause of it. Also in regard to the action of the essential oils on persons we have but little knowledge. On many persons the use of the common flavoring extracts produce very unpleasant symptoms, as thirst, acute pain and spasms of the stomach and bowels, with eructations and purging.

I will now, as briefly as possible, discuss what, in my opinion, was the true cause of the poisoning in these cases, and which I will sustain by undoubted authority. I have already referred to the dangers of hasty conclusions—the liability to be wrong, and the importance of complete analysis before expressing any opinion—and that the most learned chemists and toxicologists have made errors, which is more the evidence of an intricate science, requiring close scrutiny, than of ignorance on the part of the operator or investigator.

If you will read carefully "Taylor on Poisons," article on Lead, you will see the extreme care necessary to be taken in testing for this poison, and the very minute quantity of it necessary to produce all the ill effects of the poison, even in quantities so small as "not to be written in decimals," as was stated to be found in one of these samples of ice cream custard.

Lead, as an accidental ingredient in articles of food, may be found in liquids used for culinary purposes, especially if they contain a free acid, and are liable to become impregnated with oxide of lead, derived from the glaze of the vessel in which they are kept, and thus form poisonous salts—more especially if vinegar is used, forming the acetate of lead.

Litharge-glaze is easily dissolved by acid, alkaline or fatty substances. All newly glazed vessels yield a larger or smaller proportion of lead on boiling in them pure acetate acid, or a solution of potash free from lead. In this manner the poisonous nature of the glaze may be tested, the oxide of lead being dissolved in the acid or alkali.

Lead may be an accidental ingredient in distilled water, in the waters of the essential oils, as well as in certain medicines. Some of these acquire an impregnation of it in the process of manufacture, when leaden vessels are used in their making.

Solutions of alkaline salts, kept in flint glass bottles in the drug stores, generally contain lead. The alkalies, potash and soda, the carbonates and bicarbonates, the alkaline silicate, phosphates of soda, and some others, are thus rendered impure by the quantity of lead contained in them.

Mr. Proctor, of New York, mentions a case of some novelty in reference to the contamination of food with lead. Four men partook of a rhubarb pie and milk for supper. Shortly after, they were all seized with violent vomiting and purging, and intense thirst and colic pains. A portion of this vomitted matter and food were examined by Mr. Proctor, and lead was detected in them. The only source the lead could be traced was the litharge glaze of the pans in which the milk was kept.

Beer and cider have produced ill effects, not from "fermentation," "incipient" or otherwise, but because of the leaden pipes used in its manufacture, and being acid, is allowed to remain too long in them, acquiring an impregnation of lead. Especially may this accident happen to cider, which is a highly acid liquid, and leaden vessels are generally used in its manufacture. This should be remembered by cider drinkers, because many fatal cases have been reported from this cause. The remedies will be at once recognized if these facts are not forgotten. When a test is desired, remember that liquids of this class are affected by the oxide of lead, and may be easily discovered by being turned, more or less, of a brown color by sulphide of ammonia, or by sulphuretted hydrogen.

Pork is often poisoned by being salted in leaden vessels and allowed to remain soaking in the brine. The effect is to impregnate the pork with chloride of lead, derived from common salt (chloride of sodium).

Flour has produced fatal results in consequence of the grinding machinery of the mill being repaired with lead cement and covered with paste, the latter being worn away and the lead exposed and mixed with the flour in grinding. All millers should remember this, as it is a common thing with millers, even in balancing their rocks, to use lead. No doubt many have lost their lives from this cause, without suspicion of the source. "Taylor on Poisons" reports that during the year 1866 whole families were poisoned in one district, in New York, by using flour manufactured at a mill where the miller had been in the habit of filling up cavities in the mill stones with lead.

Salt, butter and sugar have been poisoned by lead in their preparation. Snuff and tobacco are frequently adulterated with lead, to the extent of causing symptoms of chronic poisoning. The red oxide and the chromate of lead are the compounds used, the object of the adulteration being to improve the color and the saleable value of the snuff, at the expense of the health, and may be life, of the customer. This noxious adulteration has often produced paralysis and other forms of lead poisoning to the devotee of "the stick and bottle." Out of forty-three samples of snuff examined by Dr. Harrall, of London, chromate of lead was detected in nine, and oxide of lead in twelve. This subject has been lately investigated by Dr. Meyer, of Boston, and the results were most unsatisfactory for "snuff-dippers."

Apart from this wilful adulteration, snuff and tobacco are liable to be thus impregnated from being kept in vessels and wrappers made of lead, or a spurious alloy of lead, called "patent tin-foil." It is dangerous to buy and use tobacco packed in leaden wrappers. I have spoken at length concerning snuff and tobacco poisoned with lead, because they are in such universal use; and apart from the destruction of health resulting from the pernicious effects of these articles on human health, even when pure, how much greater must be their baneful influence when poisoned by so subtle an agent as lead, and requiring such minute portions to destroy health and even life. I hope these hints and cautions will attract the attention of those who indulge "in the weed," and good may result from the discussion.

Chocolate is sometimes packed the same way; also, preserves, fruits, wines, pickles, etc., are often put up in jars and bottles in which lead and tin are employed in the form of a screw cap, as a patented substitute for corks. On examination, the contents have been found strongly impregnated with lead. In France and England these articles, thus prepared, carry with them certain penalties, and properly—where the public health is more a subject of legislation than in the "best government the world ever saw;" for in Georgia, the "Empire State," a contracted and over conscientious legislature brutally repealed the most important bill ever passed in the State for the protection of the health of its citizens. I refer to the "Board of Health." No greater error has been made in legislation. The Georgia Medical Association worked and waited patiently for this bill to become a law, and the lamented Nottingham spent his best days in impressing on the legislature and his professional brethren the importance and value of this bill; and the very last effort of his valuable life was in furnishing most valuable papers to the Board of Health on this subject, of which he died an honorable and valuable member.

To return. The rule for determining this spurious tin-foil is simple, and any physician or druggist can do it; and I would advise all persons who use these articles thus put up, to get their physician or druggist to make the test. I will give it, for convenience, though I presume all know it: Add to two ounces of water one drachm of sulphuric acid and one and a half drachms of nitric acid. Plunge a strip of the foil into this mixture. The tin is soon oxidized and removed, and the lead appears under its true color and with its usual properties. It will be thus seen if it constitutes the greater bulk of the alloy.

I might go on and discuss the impregnation of water by lead, passing through leaden pipes, but have not space or time. In all articles of diet no cause has been so fruitful of trouble as a source of poisoning, as lead. Then all lead contaminations are objectionable, and no degree of it can be considered safe. Lead is a cumulative poison, and affects some persons most powerfully in the smallest quantities, in consequence of unfortunate idiosyncracies. I feel constrained, before quitting this part of the subject, to caution the public against an evil practice which has lately sprung up, of substituting for block tin a cheap alloy of tin and lead, in the so-called tinning of iron and copper utensils.

Also, not an unfrequent cause of accidental poisoning, is the use of copper vessels for culinary purposes. All the salts of copper are poisonous. The symptoms do not appear as in lead poisoning under four or five hours, or even longer, after exposure. The symptoms are great nausea, severe colic pains, cramps and purging.

An important rule to be remembered by all housekeepers is, no acid, saline (salty), fatty or oily liquid should be prepared as an article of food in a copper vessel. (See Aund's Hyg., 1832, vol. 1, p. 102). The preparation of fruits, such as preserves, in copper vessels, is necessarily attended with great risk. Copper vessels should be lined with pure tin, but if cleanliness is adopted, the risk is greatly lessened. The wearing away of the tin must be carefully watched, as danger lurks under it. Accidental poisoning has been produced by the use of so-called German silver, which is an alloy of copper and zinc with nickel, and properly should be called white brass. When spoons of this metal present greenish-colored spots in contact with butter and vinegar, they are liable to poison.

Many of the green pickles sold in the shops are impregnated with the vegetable salts of copper (blue vitriol) for the purpose of giving them a rich green color. An easy and sure test of this fact is to immerse a bright needle (common sewing) into the pickle, when it will be speedily coated with copper. I would then advise all ladies, when they go to purchase pickles, to take a bright, polished needle and test them as above.

To conclude this already too long paper, I will repeat, that the difficulties in the way of arriving at a positively satisfactory conclusion as to the cause and nature of this poison, communicated through the ice cream consumed by the parties who were poisoned, have already been referred to, in the fact that those medical gentlemen who have expressed themselves, had all the conditions under which the cases occurred, except the most important ones, namely, "a chemical examination of the ice cream eaten and the matter vomited, and the *post mortem* appearances and chemical examination of those who died"—no opinion being worth much without these. Consequently, I have mainly attempted to meet and controvert the "germ theory" advanced by Dr. Cheatham, and slightly modified by Dr. Mattauer, both gentlemen of character and admitted skill in their profession, and to urge my own theory of the most probable cause being the lead contamination which was contained in the ice cream custard. I have sustained my position by giving the opinions of Dr. Edward R. Squibbs, of Brooklyn, New York, which is considered and acknowledged to be more valuable than any other toxicologist in America, and also has no superior in Europe. Dr. Squibbs asserts most positively, with all the facts before him, including the articles from the *Dawson Journa* and *Macon Telegraph and Messenger*, that "there is nothing in the present state of knowledge of the process of fermentation to warrant any such conclusion," (as advanced by Drs. Cheatham and Mattauer) "nor any thing to warrant the supposition that any germs formed in any such mixture (of eggs, milk and sugar) within the time mentioned, or indeed in any length of time compatible with the mixture being in an edible condition, could be poisonous, or be developed into poisonous animalculæ." Language cannot be more expressive of certainty—not a shadow of doubt, even.

I also quoted from a letter written to me on this subject by Dr. S. P. Sharples, State Assayer of Massachusetts and Professor of Chemistry and Toxicology at Harvard University, Cambridge, in which he says: "The explanation by Dr. Mattauer of a well known fact, is hardly tenable, I think," proceeding to give his reasons, too lengthy to be quoted here. He thinks the circumstances all point to the "lead theory," or contamination, as accounting for the trouble. I have quoted, also, freely from "Taylor on Poisons," a standard work on the subject, both in America and Europe, all of which, in my humble opinion, fully sustains my position on this subject. I have digressed from the proper discussion of the subject to state some practical facts, hoping thereby to instruct and caution those who take delight in the culinary department, concerning the use of certain culinary utensils of modern manufacture.

In conclusion, I will state I have expended much time and thought in the investigation of this subject during the past three months, both on account of its imperfect investigation at the time of its occurrence, and for the defense of myself as to my professional connection with the cases in the boarding house of Mrs. Gregory, growing out of the various conflicting and ridiculous reports which have been extensively circulated and freely discussed in this and other communities.

If I may have said anything to throw "more light" on this subject, or to instruct our housewives concerning the dangerous results from the use of culinary vessels of tin, copper or glazed earthenware, where a fatal poison may lurk unknown and unsuspected, ready to be brought into life and activity by contact with certain substances, I will be sufficiently compensated for the time and labor devoted to the preparation of this paper. It is a very important subject, and should attract more attention than we have been accustomed to devote to it. Then let us learn a lesson from these cases, and the constantly recurring ones weekly reported in our newspapers, and give more attention to the character of our culinary apparatus. It is no unusual thing to read in our morning paper the startling intelligence of a family suddenly attacked with severe and dangerous symptoms after partaking of a meal; but no further investigation than to determine if there is any criminal intent—especially if all recover. We do not know how many valuable lives are yearly sacrificed, and how much precious health wrecked by those insidious poisons which find their way into our food through these souroes, and are attributed to accidental or other causes, if any conclusion at all is arrived at or considered. I have no doubt but much of the paralysis and other suddenly severe diseases we so constantly hear of and are called on to treat, and which of late years is attracting so much attention from prominent physicians, if fully and carefully investigated, could be traced to the culinary department for their origin. It is well known by observing medical men, who have practiced medicine during the past twenty-five years, that in the days of our fathers and grand-fathers, when the people had only the old iron pots and skillets and other rude implements, with the old familiar "pot-racks" and open fire-places to cook their food, and when the sound of the pestle and "biscuit block" could be heard throughout the town, operated by the muscles of the old cook woman, and when our food was prepared and preserved alone by the agencies furnished by nature, that our people had stouter constitutions, and lived out their allotted days of three score and ten years, with "*sana mens in sano corpori*," and that paralysis, softening of the brain, neuralgia, and the long list of chronic maladies, whose names are now "legion," was scarcely heard of. But "*O, tempora, O, mores!*" these latter days of cooking stoves, run by steam, by gas, and by coal, with their attractive and numerous paraphernalia of patent accompaniments, all bright and shining, nickel-plated, lithrage-glazed, German silvered and white-brassed, etc., to say nothing of the modern means of preparing and preserving meats, fruits, vegetables and other articles of diet, robbing nature herself of all part in these processes. Here we find abundant cause for our manifold troubles and misery, and what an unexplored field looms up for the philanthropic and enterprising physiologist—for the industrious members of the "Boards of Health," the conservators of the public health, and for our legislators, who should care less for self-aggrandizement and emolument, and more *pro bono publico.* But this subject is too vast for discussion in a paper like this. Those who would live long and be happy, if wise, will profit by these hints.

